# Non-Norovirus Viral Gastroenteritis Outbreaks Reported to the National Outbreak Reporting System, USA, 2009–2018

**DOI:** 10.3201/eid2702.203943

**Published:** 2021-02

**Authors:** Claire P. Mattison, Molly Dunn, Mary E. Wikswo, Anita Kambhampati, Laura Calderwood, Neha Balachandran, Eleanor Burnett, Aron J. Hall

**Affiliations:** Centers for Disease Control and Prevention, Atlanta, GA, USA (C.P. Mattison, M. Dunn, M.E. Wikswo, A. Kambhampati, L. Calderwood, N. Balachandran, E. Burnett, A.J. Hall);; Cherokee Nation Assurance, Arlington, Virginia, USA (C.P. Mattison, A. Kambhampati, N. Balachandran);; Oak Ridge Institute for Science and Education, Oak Ridge, Tennessee, USA (L. Calderwood)

**Keywords:** viral gastroenteritis, astroviridae, sapovirus, rotavirus, adenoviridae, long-term care, disease outbreaks, viral diseases, viruses, United States, National Outbreak Reporting System, NORS, surveillance, foodborne diseases

## Abstract

During 2009–2018, four adenovirus, 10 astrovirus, 123 rotavirus, and 107 sapovirus gastroenteritis outbreaks were reported to the US National Outbreak Reporting System (annual median 30 outbreaks). Most were attributable to person-to-person transmission in long-term care facilities, daycares, and schools. Investigations of norovirus-negative gastroenteritis outbreaks should include testing for these viruses.

In the United States, ≈179 million cases of acute gastroenteritis (AGE) occur annually (*1*). Norovirus is the leading cause of AGE in the United States; other viral causes include adenovirus (specifically group F or types 40 and 41), astrovirus, sapovirus, and rotavirus (*2*,*3*). These viruses are spread primarily through the fecal–oral route through person-to-person contact or through contaminated food, water, or fomites (*4*–*8*).

The epidemiology of outbreaks associated with sapovirus, another calicivirus, adenovirus types 40 and 41, and astrovirus is not well understood (*6*). In addition, our understanding of rotavirus is evolving in the postvaccine era. In 2009, the Centers for Disease Control and Prevention launched the National Outbreak Reporting System (NORS), which collects information from local, state, and territorial health departments on foodborne, waterborne, and enteric disease outbreaks (*9*). To inform prevention efforts, we describe AGE outbreaks caused by adenovirus, astrovirus, sapovirus, and rotavirus that were reported to NORS during 2009–2018.

## The Study

NORS is a dynamic, voluntary outbreak reporting system. For each reported outbreak, health departments report the mode of transmission, number of confirmed and suspected cases, and aggregate epidemiologic and demographic information as available. NORS defines outbreaks as >2 cases of similar illness associated with a common exposure or epidemiologic link (*9*). Health departments determine reported outbreak etiologies on the basis of available laboratory, epidemiologic, and clinical data; specific laboratory testing protocols vary by health department. Outbreak etiologies are considered confirmed when >2 laboratory-confirmed cases are reported and considered suspected when <2 laboratory-confirmed cases are reported. Outbreaks are considered to have multiple etiologies when >1 etiology is confirmed or suspected.

Our analysis includes NORS data from outbreaks occurring during January 1, 2009–December 31, 2018 with adenovirus, astrovirus, rotavirus, or sapovirus as a confirmed or suspected etiology. NORS waterborne outbreak data were available through December 31, 2017. Data were extracted December 4, 2019.

Sex, age, symptom, and clinical outcomes percentages were calculated using the total number of cases for which information was available. Outbreak size and duration were compared by using the Kruskall–Wallis test. Analyses were performed by using SAS 9.4 (SAS Institute Inc., https://www.sas.com).

During 2009–2018, a total of 323 (1.2%) of 28,071 outbreaks reported to NORS had a reported etiology, including adenovirus, astrovirus, rotavirus, or sapovirus. A single etiology was reported in 244 (75.5%) outbreaks, of which 184 (57.0%) were confirmed (Table 1); of these 244 outbreaks, rotavirus accounted for 123 (50.4%), sapovirus for 107 (43.9%), astrovirus for 10 (4.1%), and adenovirus for 4 (1.6%). Multiple etiologies were reported in 79 (24.5%) of the 323 outbreaks; 51 (64.5%) of the 79 also included norovirus as an etiology. The most common etiology combinations were rotavirus and norovirus (19 [24.1%]), sapovirus and norovirus (7 [8.9%]), and sapovirus, norovirus, and astrovirus (7 [8.9%]).

**Table 1:** Summary of outbreak characteristics, by suspected or confirmed outbreak etiology, for outbreaks attributable to adenovirus, astrovirus, rotavirus, or sapovirus, National Outbreak Reporting System, USA, 2009–2018*

Characteristic	Etiology					
	Adenovirus	Astrovirus	Rotavirus	Sapovirus	All single-etiology	Multiple etiologies†
No. outbreaks						
Total	4	10	123	107	244	79
Annual median	0	1	9	12	26	4
States or territories	3	6	28	22	32	22
Confirmed or suspected						
Confirmed	3 (75)	5 (50)	70 (56.9)	70 (65.4)	148 (60.7)	29 (36.7)‡
Suspected, 1 positive	1 (25)	5 (50)	30 (24.4)	25 (23.4)	61 (25.0)	36 (45.6)‡
Suspected, 0 positives	–	–	23 (18.7)	12 (11.2)	35 (14.3)	14 (17.7)‡
Median duration, d (range)	19 (6–39)	11 (1–22)	11 (1–39)	9 (1–65)	10 (1–65)	18 (1–121)
Mode of transmission						
Person-to-person	3 (75)	6 (60)	104 (84.5)	77 (72)	190 (77.9)	65 (82.3)
Foodborne	–	1 (10)	4 (3.3)	15 (14)	20 (8.2)	4 (5.1)
Waterborne	1 (25)	–	–	–	1 (0.4)	–
Indeterminate or unknown	–	3 (30)	15 (12.2)	15 (14)	33 (13.5)	10 (12.6)
Setting of exposure						
Long-term care facility	–	2 (20)	80 (65.0)	63 (58.9)	145 (59.4)	26 (32.9)
Child daycare	–	2 (20)	19 (15.4)	6 (5.6)	27 (11.1)	28 (35.4)
School or university	1 (25)	3 (30)	4 (3.3)	12 (11.2)	20 (8.2)	10 (12.7)
Restaurant or catering	–	1 (10)	2 (1.6)	13 (12.1)	16 (6.6)	2 (2.5)
Healthcare facility	1 (25)	1 (10)	1 (0.8)	1 (0.9)	4 (1.6)	3 (3.8)
Other, indeterminate, or missing	2 (50)	1 (10)	17 (13.8)	12 (11.2)	32 (13.1)	10 (12.7)
Year						
2009	–	–	8 (6.5)	2 (1.9)	10 (4.1)	1 (1.3)
2010	–	–	7 (5.7)	–	7 (2.9)	1 (1.3)
2011	–	1 (10)	6 (4.9)	3 (2.8)	10 (4.1)	4 (5.1)
2012	1 (25)	1 (10)	5 (4.1)	10 (9.3)	17 (7.0)	3 (3.8)
2013	–	–	10 (8.1)	12 (11.2)	22 (9.0)	4 (5.1)
2014	–	–	10 (8.1)	21 (19.6)	31 (12.7)	3 (3.8)
2015	1 (25)	2 (20)	37 (30.1)	13 (12.1)	53 (21.7)	6 (7.6)
2016	–	4 (40)	8 (6.5)	18 (16.8)	30 (12.3)	8 (10.1)
2017	1 (25)	1 (10)	16 (13.0)	16 (15.0)	34 (13.9)	25 (31.6)
2018	1 (25)	1 (10)	16 (13.0)	12 (11.2)	30 (12.3)	24 (30.4)

*Values are no. (%) unless otherwise indicated. –, no outbreak reported. †Outbreaks attributable to adenovirus (14), astrovirus (15), rotavirus (34), or sapovirus (33) along with >1 other etiology. The most common combinations reported were rotavirus and norovirus (19 outbreaks); sapovirus and norovirus (7 outbreaks); sapovirus, norovirus, and astrovirus (7 outbreaks); and adenovirus and norovirus (4 outbreaks). ‡Confirmed or suspected for >1 of the 4 viruses of interest. Suspected (1 positive) outbreaks include 7 outbreaks where 1 viral etiology of interest was confirmed and another viral etiology was suspected, with a single positive result.

A median 30 outbreaks were reported per year (range 8–59 outbreaks). Reporting increased over time; most (62.0%) multiple-etiology outbreaks were reported during 2017–2018 (Table 1). Outbreaks were reported by 31 states and Puerto Rico; 5 states (Wisconsin [63 (19.5%)], Oregon [51 (15.8%)], Ohio [31 (9.6%)], Virginia [19 (5.9%)], and Illinois [19 (5.9%)]) accounted for >50% of reports. Subsequent results are presented for single-etiology outbreaks only.

**Figure 1:**
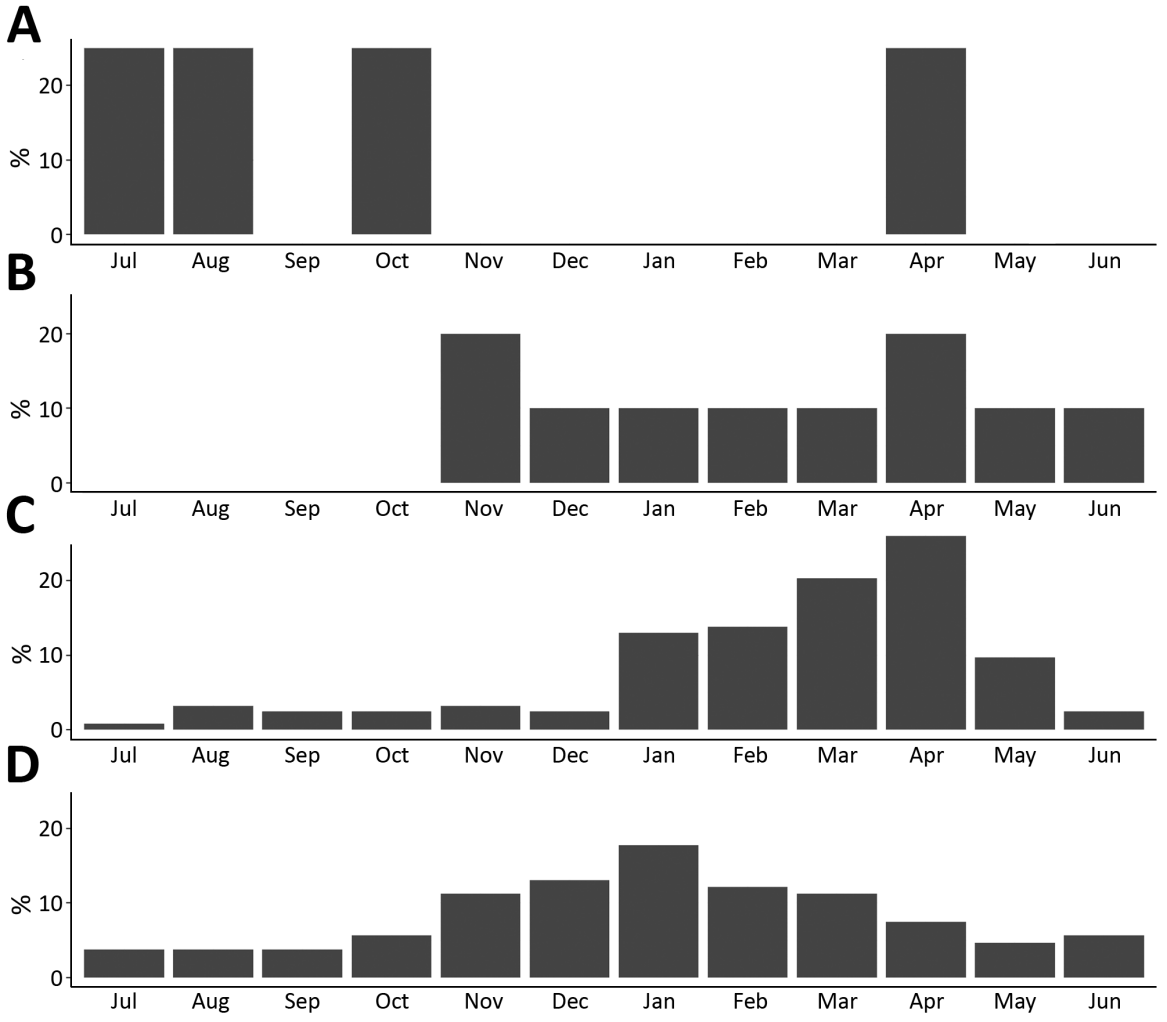
Percentage of outbreaks reported per month, by suspected or confirmed outbreak etiology, for single-etiology outbreaks attributable to adenovirus (A), astrovirus (B), rotavirus (C), or sapovirus (D), National Outbreak Reporting System, USA, 2009–2018.

Median outbreak size (17 cases [p = 0.62]) (Table 2) and outbreak duration (10 days [p = 0.30]) (Table 1) did not differ between etiologies. Most astrovirus (8 [80%]) and sapovirus (78 [72.9%]) outbreaks occurred during November–April. Most rotavirus outbreaks occurred during January–May (102 [82.9%]) (Figure 1).

**Table 2:** Summary of outbreak case characteristics by suspected or confirmed outbreak etiology for outbreaks reporting adenovirus, astrovirus, rotavirus, or sapovirus — National Outbreak Reporting System, 2009–2018*

Characteristic	Etiology					
	Adenovirus	Astrovirus	Rotavirus	Sapovirus	All single-etiology	Multiple etiologies†
No. outbreak cases						
Total	83	275	2,526	3,112	5,996	5,278
Median	19	20.5	16	17	17	18
Range	13–32	9–84	2–82	2–528	2–528	2–2,274
Sex						
No. cases with information	82	162	1,750	1,694	3,688	4,070
F	36 (43.9)	98 (60.5)	1,171 (66.9)	1,062 (62.7)	2,367 (64.2)	2,365 (58.1)
M	46 (56.1)	64 (39.5)	579 (33.1)	632 (37.3)	1,321 (35.8)	1,705 (41.9)
Age group, y						
No. cases with information	40	142	1,161	2,071	3,414	3,676
<1	0	3 (2.1)	47 (4.0)	3 (0.1)	53 (1.6)	109 (3.0)
1–4	1 (2.5)	17 (12.0)	222 (19.1)	75 (3.6)	314 (9.2)	375 (10.2)
5–9	7 (17.5)	27 (19.0)	185 (15.9)	350 (16.9)	569 (16.7)	1,817 (49.4)
10–49	17 (42.5)	66 (46.5)	221 (19)	854 (41.2)	1,158 (33.9)	1,034 (28.1)
>50	15 (37.5)	29 (20.4)	487 (42.0)	789 (38.1)	1,320 (38.7)	340 (9.3)
Clinical outcome‡						
Hospitalization	21/71 (29.6)	0/221 (0)	87/2,030 (4.2)	17/2,154 (0.8)	125/4,476 (2.8)	44/4,189 (1.1)
Death	2/71 (2.8)	0/275 (0)	8/2,127 (0.4)	2/2,283 (0.09)	12/4,756 (0.25)	5 /4,273 (0.1)

*Values are no. (%) unless otherwise indicated. †Outbreaks attributable to adenovirus (14), astrovirus (15), rotavirus (34), or sapovirus (33) along with >1 other etiology. The most common combinations reported were rotavirus and norovirus (19 outbreaks); sapovirus and norovirus (7 outbreaks); sapovirus, norovirus, and astrovirus (7 outbreaks); and adenovirus and norovirus (4 outbreaks). ‡No. cases/no. cases with data available (%).

The most common modes of transmission were person-to-person (190 [77.9%]), indeterminate or unknown (33 [13.5%]), and foodborne (20 [8.2%]) (Table 1). Most foodborne outbreaks were attributable to sapovirus (15 [75.0%]). Common outbreak settings included long-term care facilities (LTCFs) (145 [59.4%]), child daycares (27 [11.1%]), and schools (20 [8.2%]) (Table 1). Most rotavirus (80 [65.0%]) and sapovirus (63 [58.9%]) outbreaks occurred in LTCFs.

Among 3,688 cases for which data were available, 64.2% were in women and girls. Cases occurred among all age groups (Table 2). Compared with 20.4% of astrovirus outbreak cases, higher percentages (37.5%–42.0%) of adenovirus, rotavirus, and sapovirus outbreak cases were among persons >50 years old. Rotavirus outbreaks had the highest proportion of cases in children <1 year old (4.0%) and 1–4 years old (19.1%).

**Figure 2:**
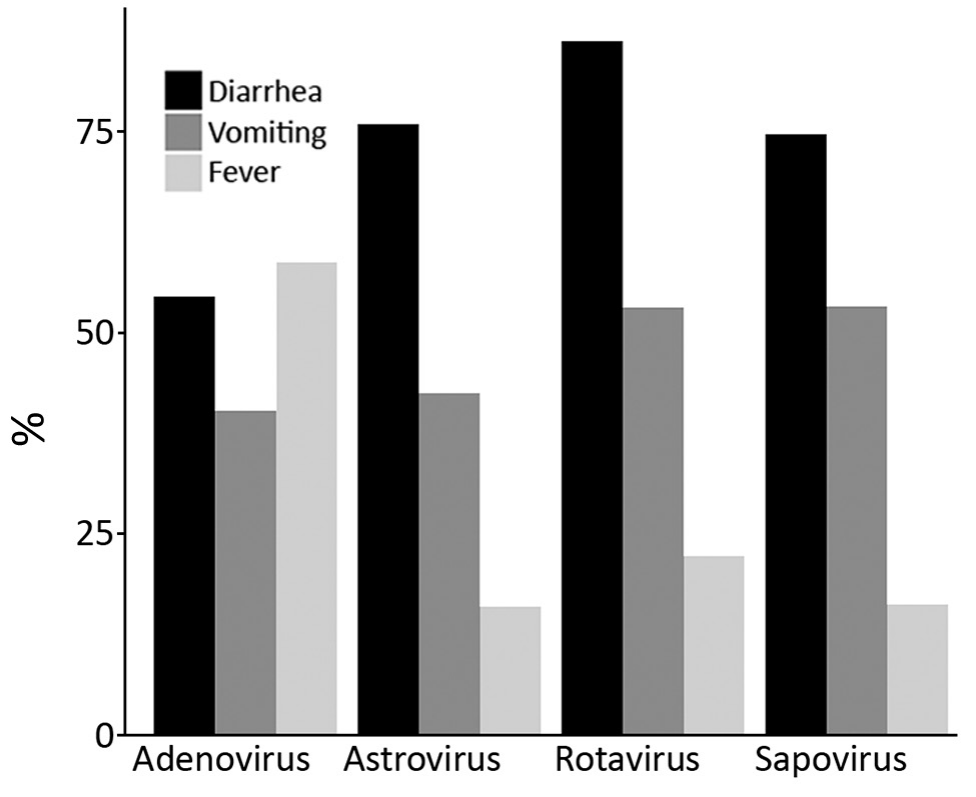
Percentage of cases with symptom information including diarrhea, vomiting, and fever, by suspected or confirmed outbreak etiology, for single-etiology outbreaks attributable to adenovirus, astrovirus, rotavirus, or sapovirus, National Outbreak Reporting System, USA, 2009–2018.

Among adenovirus outbreaks, 58.8% of case-patients reported fever, 54.5% reported diarrhea, and 40.4% reported vomiting. Across the other 3 viral etiologies, diarrhea was the most reported symptom, followed by vomiting and fever (Figure 2). Bloody stools were reported for <2% of case-patients (data not shown). Adenovirus outbreaks were responsible for the highest proportions of hospitalized case-patients (21 [29.6%]) and deaths (2 [2.8%]) (Table 2).

## Conclusions

During 2009–2018, a total of 323 outbreaks caused by adenovirus, astrovirus, rotavirus, or sapovirus were reported to NORS. These 4 viral pathogens typically cause mild, self-limiting illness, as evidenced by the low reported hospitalization and case-fatality rates. In adenovirus outbreaks, >25% of case-patients were hospitalized and >50% reported fever, but because of the low number of outbreaks reported, these characteristics are likely not representative of all enteric adenovirus infections. Like norovirus outbreaks, astrovirus and sapovirus outbreaks often occurred in closed settings, were mostly transmitted through person-to-person contact or foodborne transmission, and had winter seasonality (*6*,*10*).

In the United States, rotavirus vaccination has substantially reduced incidence in younger, vaccinated populations and indirectly benefitted older, unvaccinated populations (*11*). Reported rotavirus outbreaks affected both young and older populations, and most occurred in LTCFs. Like other viral AGE etiologies, rotavirus is most often transmitted through person-to-person contact, spreads easily in closed settings, and most commonly causes diarrhea and vomiting. Sapovirus, astrovirus, and rotavirus should thus be considered in outbreaks initially suspected to be norovirus where case-patients have negative results.

All 4 viruses discussed in this report have low infectious doses, are shed asymptomatically and postsymptomatically, and can survive on surfaces, facilitating transmission in closed or semi-closed settings (*4*–*6*,*8*,*12*). Existing viral AGE outbreak prevention and control recommendations (i.e., handwashing, surface disinfection with appropriate products [e.g., bleach-based cleaners], exclusion of symptomatic persons from daycare, school, or work and food preparation for others until 48 hours after symptoms resolve [*13*]) are useful against viral AGE of all etiologies.

Many viral gastrointestinal outbreaks go unreported, and determination of outbreak etiology varies based on testing availability; adenovirus, astrovirus, and sapovirus testing only recently became widely available through multipathogen test panels (*14*). In 2012, the Centers for Disease Control and Prevention ­established the Unexplained Viral Diarrhea network in partnership with the California, Minnesota, and Oregon state public health laboratories to comprehensively test stool specimens from norovirus-negative outbreaks to better understand the burden of these viruses (*15*). This network partially explains the geographic heterogeneity of outbreak reports in NORS; as such, the observed geographic variability is most likely attributable to differences in testing and reporting practices, not actual differences in incidence.

Multiple etiology outbreaks were reported more often in recent years, likely because of increased availability of multipathogen test panels. Multiple-etiology outbreaks involving adenovirus, astrovirus, rotavirus, or sapovirus were commonly found in combination with norovirus. Further study is needed to determine whether each of these detected pathogens contributed to outbreak illnesses or represent detection of asymptomatic shedding.

Adenovirus, astrovirus, rotavirus, and sapovirus remain important causes of AGE outbreaks in the United States and should be considered as potential etiologies, especially for norovirus-negative outbreaks. More widespread testing and reporting will help to advance understanding of the burden and epidemiology of these viruses.
